# What Drives Nutritional Supplement Use Among Academics? An Intention–Behavior Model of Motivation, Work Stress, and Digital Influences

**DOI:** 10.3390/ijerph23050629

**Published:** 2026-05-09

**Authors:** Şermin Önem

**Affiliations:** E-Commerce and Marketing Program, Vocational School, Istanbul Atlas University, Istanbul 34408, Türkiye; sermin.onem@atlas.edu.tr

**Keywords:** health motivation, academic work stress, digital health literacy, intention–behavior, nutritional supplement use

## Abstract

**Highlights:**

**Public health relevance—How does this work relate to a public health issue?**

- Nutritional supplement use represents a growing health-related consumption behavior shaped by motivational and occupational factors in digitally mediated environments.
- Examining academics as a distinct, highly educated segment provides insight into how work stress and health motivation influence preventive health behaviors.

**Public health significance—Why is this work of significance to public health?**

- The study demonstrates that intrinsic health motivation and academic work stress significantly drive supplement usage intention, whereas digital health literacy and digital marketing influence do not exert direct effects.
- The findings clarify the intention–behavior mechanism underlying supplement consumption among knowledge-intensive professionals.

**Public health implications—What are the key implications or messages for practitioners, policy makers and/or researchers in public health?**

- Public health strategies should address occupational stress and intrinsic motivation when promoting rational and evidence-based supplement use.
- Interventions targeting supplement consumption should not assume that digital literacy alone ensures informed health decisions among highly educated populations.

**Abstract:**

Nutritional supplement use has emerged as an important health-related consumption behavior in digitalized environments, with implications for both public health and individual well-being. While prior research has largely focused on general populations, limited attention has been paid to how occupational pressures and digital information contexts jointly shape supplement-related decision-making among highly educated professionals. Addressing this gap, this study examines the behavioral determinants of nutritional supplement use among academics within an intention–behavior framework. Using survey data collected from academic professionals, the proposed model was tested through confirmatory factor analysis and structural equation modeling. The findings reveal that health motivation and academic work stress significantly predict supplement use intention, which, in turn, strongly influences actual consumption behavior. In contrast, digital health literacy and digital marketing exposure do not exert significant direct effects on usage intention. These results provide theoretical insight into the boundary conditions of informational determinants in consumer behavior models, suggesting that intrinsic motivation and contextual stressors may play a more dominant role than digital influences among highly educated consumers. From a practical perspective, the findings highlight the importance of addressing stress-related health coping mechanisms and motivation-driven behaviors in promoting informed supplement use.

## 1. Introduction

Nutritional supplement consumption has increasingly expanded beyond clinical and athletic contexts into everyday professional life, emerging as prominent preventive health behavior within modern work environments. Nutritional supplements are commonly defined as non-prescription health products containing vitamins, minerals, amino acids, and other bioactive compounds intended to complement dietary intake and support general well-being [1,2,3]. Rapid lifestyle changes have intensified occupational demands, and growing engagement in self-care practices have substantially accelerated supplement adoption across populations [2,3,4,5]. Contemporary public health research increasingly frames supplement use as a form of preventive self-care behavior embedded within broader health-oriented lifestyles, rather than as isolated consumption decisions driven by short-term promotional stimuli [2,6,7,8].

Modern professional life is characterized by sedentary routines, time scarcity, and sustained cognitive and emotional demands, which have been shown to significantly influence health-related coping behaviors. Empirical evidence suggests that occupational stress, limited opportunities for balanced nutrition, and heightened workload pressures contribute to increased reliance on nutritional supplements as compensatory health-management strategies [3,5,8]. At the same time, substantial cross-national variation in supplement use highlights the importance of socio-economic conditions, education, and health awareness in shaping preventive health behaviors. While existing studies have largely focused on general population trends, comparatively little is known about how highly educated professionals navigate supplement-related decisions within demanding occupational contexts. Academic professionals represent a particularly relevant group, characterized by intensive workloads, continuous information exposure, and proactive health management tendencies, making them a valuable population for understanding behavioral determinants of preventive health practices.

While prior studies have provided valuable insights into general patterns of supplement use, less is known about how highly educated consumer groups navigate supplement-related choices within demanding occupational environments. In particular, academics represent a unique segment characterized by high cognitive demands, intensive workloads, and frequent exposure to digital health information, making them a theoretically and practically relevant group for examining health-related consumption behavior.

Within this context, nutritional supplement use can be understood as a form of intentional consumer behavior shaped by both individual motivations and situational pressures. Understanding how consumers develop usage intentions and translate these intentions into actual behavior therefore remains a central concern in consumer behavior research. In increasingly complex professional and health-related consumption contexts, internal motivational states and situational pressures may play a more decisive role in shaping intention–behavior processes.

Within intention-based consumer behavior models, intention is widely recognized as the most proximal antecedent of behavior [9,10]. The Theory of Planned Behavior posits that intention emerges from the interplay of personal attitudes, subjective norms, and perceived behavioral control, serving as a direct precursor to observable behavior [9]. However, intention formation itself is influenced by a variety of psychological, contextual, and informational determinants. Prior research has frequently emphasized informational factors—such as literacy, knowledge, and exposure to marketing messages—as key drivers of consumer intention [11,12,13]. Yet, these factors may function differently across consumer segments. Among highly educated consumers, informational competence may represent a baseline condition rather than an active determinant, thereby reducing its explanatory power in intention formation. This calls for a more nuanced understanding of the relative importance of motivation, stress, and information in contemporary consumer decision-making.

One motivational construct that has received increasing attention in consumer research as a central driver of health-related decision-making is health motivation [8,14]. Health motivation captures individuals’ internal desire to protect and enhance their personal well-being and plays a significant role in shaping engagement in health-oriented consumption practices [14]. Consumers with higher health motivation are more likely to actively seek solutions that align with their personal goals and values, making health motivation a critical psychological driver in intention formation. From a consumer behavior perspective, such intrinsic motivation is expected to exert a strong influence on usage intention, particularly in contexts where consumption decisions are closely tied to personal well-being.

In addition to motivational factors, contextual stressors represent an important yet underexplored determinant of consumer intention [15,16]. Academic work stress, as a form of situational pressure, may shape consumption decisions by increasing consumers’ perceived need for supportive or efficiency-enhancing solutions. When consumers experience elevated levels of stress, they may become more inclined to adopt tools that promise relief, efficiency, or control [15,17]. Although stress has traditionally been examined as an outcome variable in consumer and organizational research, its role as an antecedent of usage intention remains relatively underdeveloped in consumer behavior literature.

By contrast, digital health literacy and digital marketing influence have often been treated as central explanatory variables in digital consumption studies. Digital health literacy refers to consumers’ ability to access, understand, and apply digital health-related information to make informed health decisions [11,12], while digital marketing influence captures the persuasive impact of promotional messages and digital communication strategies on consumer perceptions, attitudes, and behavioral intentions [13,18]. Although both constructs are theoretically relevant, their effectiveness may be contingent on consumer characteristics and contextual conditions [19,20]. In samples characterized by high educational attainment, consumers are more likely to engage in systematic information processing and activate persuasion knowledge, relying less on external informational and marketing cues and more on internal judgment and motivational goals, which may weaken the direct effects of these factors on intention formation.

Despite the extensive literature on consumer intention and behavior, few studies have simultaneously examined motivational, contextual, and informational determinants within a single integrative framework [9,10,21]. Moreover, limited empirical research has investigated how these factors operate among highly educated consumer segments, where traditional informational and marketing-based explanations may be less effective [19,20]. Addressing this gap, the present study develops and empirically tests a comprehensive model that examines the effects of health motivation, academic work stress, digital health literacy, and digital marketing influence on usage intention, as well as the relationship between usage intention and actual usage behavior among academics.

Grounded in consumer behavior and intention-based theoretical frameworks [9,22], this study proposes that intrinsic motivation and situational stressors may play a more prominent role in intention formation than informational or promotional influences within highly educated professional groups [14,23]. By integrating motivational, contextual, and informational determinants within a unified framework, this study contributes to the consumer behavior literature by providing a more nuanced understanding of the drivers of nutritional supplement consumption in professional contexts.

This study contributes to literature in several ways. First, it extends the understanding of nutritional supplement consumption by examining motivational, contextual, and informational determinants within a unified intention–behavior framework [14]. Second, the study focuses on academics as a highly educated professional group, a population that has received limited attention in prior research on supplement use [3]. Third, by integrating health motivation, academic work stress, digital health literacy, and digital marketing influence within a single structural model, the study provides new insights into how different drivers shape preventive health-related consumption behaviors.

Accordingly, this study aims to examine the behavioral determinants of nutritional supplement use among academics by investigating the roles of health motivation, academic work stress, digital health literacy, and digital marketing influence in shaping usage intention and actual supplement consumption behavior within an intention–behavior framework. Building on these contributions, the following section develops the hypotheses and presents the conceptual framework of the study.

Despite the growing body of research on health-related consumption, existing studies have predominantly examined nutritional supplement use either within general populations or from a purely informational perspective. Limited attention has been paid to how motivational, occupational, and digital factors jointly shape supplement-related decision-making, particularly among highly educated professional groups. Moreover, the interplay between intrinsic health drivers and work-related stress within an intention–behavior framework remains underexplored in the literature.

Addressing this gap, the present study develops and empirically tests an integrative model that simultaneously examines the roles of health motivation, academic work stress, digital health literacy, and digital marketing influence in shaping supplement use intention and subsequent behavior among academics. By focusing on a cognitively sophisticated consumer segment, the study provides new insights into the boundary conditions of informational determinants and extends existing consumer behavior models.

## 2. Hypothesis Development

The proposed hypotheses are grounded in intention–behavior frameworks and consumer decision-making perspectives, which suggest that behavioral intentions and subsequent actions are shaped by the interplay of individual motivations, contextual stressors, and informational influences [9,10,14,15].

### 2.1. Health Motivation and Usage Intention

Health motivation refers to an individual’s intrinsic drive to maintain or enhance personal well-being and has long been recognized as a key psychological determinant of health-related consumption behaviors. From a consumer behavior perspective, intrinsically motivated consumers tend to engage in decision-making processes that are aligned with their personal goals, values, and long-term well-being rather than being driven solely by external cues [24]. In line with the Theory of Planned Behavior, individuals who hold favorable health-related attitudes are more likely to develop strong behavioral intentions [9,16]. Health motivation reflects a positive evaluative orientation toward health-promoting behaviors, and therefore, serves as a key attitudinal driver of usage intention [25].

Prior research suggests that when consumers perceive a consumption option as relevant to their health-related goals, they are more likely to form favorable intentions toward its use [8,14]. Health motivation increases consumers’ attentiveness to solutions that promise personal benefit and self-improvement, thereby strengthening intention formation [14]. In contexts where usage decisions are closely tied to well-being, health motivation is expected to serve as a primary driver of intention.

**H1.**  *Health motivation has a positive effect on usage intention.*

### 2.2. Academic Work Stress and Usage Intention

Academic work stress represents a contextual factor that may significantly shape consumer decision-making. Stressful work environments increase perceived demands and reduce individuals’ available cognitive and emotional resources, prompting consumers to seek solutions that facilitate coping, efficiency, or relief [15,26]. From a consumer behavior standpoint, stress can therefore act as a situational trigger that intensifies the perceived need for supportive products or services. According to the Theory of Planned Behavior, contextual pressures and perceived control conditions shape individuals’ intention formation [9]. Academic work stress thus represents a situational constraint that may strengthen usage intention toward health-supportive consumption.

Although stress has traditionally been examined as an outcome variable, emerging evidence suggests that contextual pressures may also function as antecedents of consumption-related intentions. When individuals experience heightened levels of work-related stress, they may become more inclined to adopt tools that promise to mitigate strain or enhance performance. As such, academic work stress is expected to positively influence usage intention.

**H2.**  *Academic work stress has a positive effect on usage intention.*

### 2.3. Digital Health Literacy and Usage Intention

Digital health literacy refers to individuals’ capacity to locate, comprehend, and critically appraise health-related information obtained through digital platforms [11,12,27]. From a theoretical perspective, greater levels of digital literacy enhance consumers’ ability to engage in informed health decision-making and to manage perceived uncertainty surrounding digital health solutions. As a result, digital health literacy has been frequently positioned as an important antecedent of behavioral intention within technology-enabled consumption environments.

However, the impact of digital health literacy may vary depending on consumer characteristics. Among highly educated consumers, digital literacy levels may be relatively homogeneous, potentially limiting its direct influence on intention formation. In such cases, literacy may function as a prerequisite for participation rather than an active motivational driver. Despite this possibility, digital health literacy remains theoretically relevant and warrants empirical examination.

Within the Theory of Planned Behavior framework, behavioral beliefs derived from information exposure shape intention formation [9]. Digital health literacy provides a key informational input that may influence consumers’ evaluation of supplement use and subsequent intention, although this effect may weaken when evaluative competence becomes a baseline condition among highly educated consumers.

**H3.**  *Digital health literacy has a positive effect on usage intention.*

### 2.4. Digital Marketing Influence and Usage Intention

Digital marketing influence refers to the persuasive effect of promotional messages, online campaigns, and digital communication strategies on consumer perceptions and decisions. Marketing communications play a central role in shaping consumer awareness, attitudes, and preferences across consumption contexts [18,28] and prior studies have consistently demonstrated their effectiveness in influencing behavioral intentions in digital environments [13,29].

Nevertheless, the influence of digital marketing may be contingent on the target audience. Consumers with high levels of education and professional expertise may rely more heavily on personal judgment, intrinsic motivation, and experiential knowledge rather than external promotional cues. Despite these potential boundary conditions, digital marketing influence remains an important factor in consumer behavior research and merits empirical testing.

Consistent with the Theory of Planned Behavior, exposure to persuasive communication contributes to the formation of behavioral beliefs that shape intention [9]. Digital marketing messages therefore represent informational stimuli that may influence consumers’ evaluation of nutritional supplements and subsequent usage intention, although their impact may diminish among cognitively sophisticated consumer segments.

**H4.**  *Digital marketing influence has a positive effect on usage intention.*

### 2.5. Usage Intention and Actual Usage Behavior

Usage intention is widely regarded as the most proximal determinant of actual behavior in intention-based consumer behavior models [9]. Intention reflects individuals’ readiness and willingness to perform a specific behavior and serves as the immediate antecedent to action. Extensive empirical evidence demonstrates that stronger behavioral intentions are consistently associated with a higher likelihood of subsequent behavior enactment [10,22].

Consistent with the Theory of Planned Behavior, behavioral intention is the most proximal determinant of actual behavior [9]. Stronger usage intention is therefore expected to translate into higher levels of actual supplement use.

In digital consumption contexts, usage intention reflects consumers’ commitment to engage with a given solution, translating psychological and contextual antecedents into observable behavior. Consistent with established consumer behavior theories, usage intention is therefore expected to exert a direct and positive influence on actual usage behavior.

**H5.**  *Usage intention has a positive effect on actual usage behavior.*

**Figure 1:**
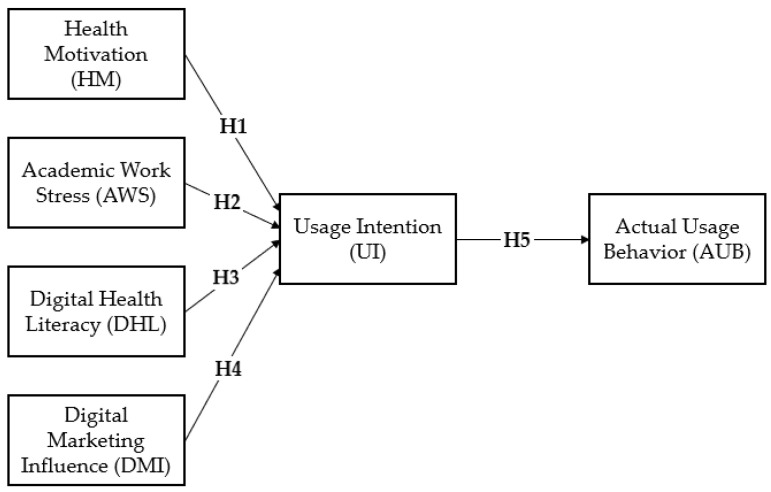
Conceptual research model. Note: The figure presents the conceptual model of the study, depicting the hypothesized relationships among the key constructs examined within the intention–behavior framework.

Taken together, these hypotheses form an integrated conceptual framework that explains nutritional supplement consumption within an intention–behavior perspective. Figure 1 summarizes the proposed hypotheses and illustrates the conceptual relationships among the study variables within the intention–behavior framework.

Figure 1 illustrates the proposed conceptual framework and hypothesized relationships among the study constructs. The model integrates motivational (health motivation), contextual (academic work stress), and informational (digital health literacy and digital marketing influence) antecedents to explain nutritional supplement usage intention and its translation into actual usage behavior within an intention–behavior framework.

## 3. Methodology

### 3.1. Sample and Data Collection

The study employed an online questionnaire to gather responses from academics affiliated with universities across Türkiye. In this study, the term “academics” refers to individuals employed in higher education institutions, including faculty members and researchers across different academic ranks (e.g., assistant professors, associate professors, professors, and research staff). Academics were selected as the target population due to their high educational attainment, intensive cognitive workload, and frequent exposure to digital health-related information, making them a theoretically relevant consumer segment for examining nutritional supplement consumption. The survey link was distributed through academic networks and institutional communication channels, and participation was voluntary and anonymous. The questionnaire was administered using Google Forms, and data were collected between December 2025 and February 2026.

Prior to conducting the main statistical analyses, the dataset was screened for missing values, outliers, and response inconsistencies to ensure the quality and reliability of the data. The cleaned dataset was then used for confirmatory factor analysis (CFA) and structural equation modeling (SEM).

This sample size was considered adequate for covariance-based structural equation modeling, as methodological guidelines indicate that SEM models with multiple latent constructs generally require sample sizes exceeding 200 observations to ensure stable parameter estimation [30,31].

Ethical approval for this study was obtained from the Social and Human Sciences Research Ethics Committee of Istanbul Atlas University (Decision No: 10, 10 December 2025). Participation in the study was voluntary and anonymous.

A total of 303 usable responses were obtained and included in the final analysis. The sample comprised academics from a wide range of universities and academic ranks, ensuring diversity in professional experience and occupational context. Respondents provided information regarding their demographic characteristics (e.g., gender, age, and academic title), as well as their current nutritional supplement usage. Importantly, supplement use was measured as actual behavior rather than hypothetical intention, allowing the study to capture real consumption patterns. Multiple types of nutritional supplements could be reported, reflecting the realistic and non-exclusive nature of supplement use.

Although individual university names were not coded, responses were obtained from academics affiliated with 108 different universities across Türkiye, ensuring substantial institutional diversity within the sample.

To ensure both geographical and institutional diversity, academics from multiple higher education institutions across different provinces in Türkiye were systematically contacted, with a minimum of two participants representing each institution. Table 1 summarizes participants’ demographic attributes alongside their nutritional supplement usage patterns. The sample included academics from both public and foundation universities across Türkiye, representing a wide range of academic titles and age groups, and participants also reported the types of nutritional supplements they currently use.

### 3.2. Measures

All study variables were operationalized using established multi-item measurement scales drawn from previous literature and adapted to reflect nutritional supplement consumption among academic professionals. Unless indicated otherwise, responses were recorded on five-point Likert scales ranging from 1 (strongly disagree) to 5 (strongly agree), with higher values representing greater intensity of the respective constructs.

The measurement items used in this study were adapted from previously validated scales in line with the conceptual scope of the research. As the questionnaire was administered in Turkish, the original English items were first carefully reviewed, translated, and contextually adapted to reflect nutritional supplement use among academics. During this process, minor modifications were made to item wording to ensure relevance to the study context while preserving the theoretical meaning of the original constructs. To ensure content validity and linguistic equivalence, the translated items were further evaluated through expert review and pilot testing. Feedback obtained from subject-matter experts and a small group of respondents was incorporated to improve clarity, wording, and contextual appropriateness prior to the main data collection. For transparency, Table 1 presents the constructs, number of items, original sources, and the rationale for adaptation.

Health Motivation was measured using four items assessing individuals’ intrinsic motivation to maintain or enhance their health. The scale demonstrated excellent internal consistency (Cronbach’s α = 0.911).

Academic Work Stress was assessed with four items capturing perceived work-related strain and exhaustion within the academic context. The scale demonstrated good internal consistency (Cronbach’s α = 0.826).

Digital Health Literacy was measured using four items assessing individuals’ capacity to obtain, interpret, and appraise health-related information from digital platforms. One negatively phrased statement was reverse coded prior to analysis. The internal consistency of the scale was adequate for exploratory research purposes (Cronbach’s α = 0.681).

Digital Marketing Influence was assessed through four items reflecting the perceived influence of online promotional content on supplement-related decision-making. The scale demonstrated strong reliability (Cronbach’s α = 0.824).

Usage Intention was measured with four items assessing participants’ intentions to use nutritional supplements. The scale demonstrated high internal consistency (Cronbach’s α = 0.886).

Actual Usage Behavior was assessed using four items capturing the frequency and consistency of nutritional supplement use. The scale showed good internal consistency (Cronbach’s α = 0.799).

Composite mean scores were computed for each construct by averaging the corresponding items. This approach is widely used in structural equation modeling to represent latent constructs in a parsimonious and interpretable manner [31], where higher mean values indicate higher levels of the respective construct.

During the initial scale development phase, several additional constructs were considered; however, only those variables that were theoretically aligned with the final research model and demonstrated empirical relevance were retained for the main analysis, ensuring parsimony and conceptual clarity in the structural model [30,31].

The full list of measurement items used in the study is provided in Appendix A.

### 3.3. Data Analysis

All statistical analyses were conducted using IBM SPSS Statistics (version 25) and IBM SPSS AMOS (version 23). Descriptive statistics and Pearson correlation analyses were first performed to examine the distributions of the study variables and explore their interrelationships. Correlation coefficients were reviewed to detect potential multicollinearity issues, and all values remained below the conservative cutoff level, indicating that multicollinearity was not a concern and that the dataset was appropriate for subsequent structural equation modeling.

Structural equation modeling (SEM) was employed as it enables the simultaneous examination of multiple relationships among latent constructs, allowing for a comprehensive evaluation of direct effects within the proposed research model. SEM is widely recommended for analyzing complex theoretical models involving interdependent relationships among variables [30,31,32].

The analytical procedure followed a two-stage structural equation modeling approach commonly adopted in consumer behavior research. In the initial stage, confirmatory factor analysis (CFA) was applied to evaluate the measurement model and assess construct reliability and validity. Model adequacy was examined using a range of fit indices, including the chi-square statistic and its ratio to degrees of freedom, the Comparative Fit Index (CFI), the Tucker–Lewis Index (TLI), and the Root Mean Square Error of Approximation (RMSEA). Given the sensitivity of chi-square to sample size and model complexity, greater emphasis was placed on relative fit indicators.

To assess the potential impact of common method bias, Harman’s single-factor test was conducted. All measurement items were included in an unrotated exploratory factor analysis. The results indicated that the first factor accounted for 37.286% of the total variance, which is below the commonly accepted threshold of 50%. This suggests that common method bias is unlikely to pose a serious threat to the validity of the findings.

The measurement model results, including composite reliability and average variance extracted values, are presented in Table 2.

Convergent validity was further assessed through Composite Reliability (CR) and Average Variance Extracted (AVE). CR values above 0.70 indicate satisfactory internal consistency, while AVE values of 0.50 or higher suggest that a construct explains more than half of the variance of its indicators [31]. Although some AVE values were slightly below the conventional 0.50 threshold, the corresponding CR values exceeded the recommended level, indicating acceptable convergent validity in line with established SEM guidelines. Overall, the results demonstrate acceptable levels of internal consistency and convergent validity.

In the final stage, the structural model was estimated to examine the hypothesized relationships among the latent variables. Standardized path coefficients and significance levels were used to evaluate the proposed hypotheses. Usage intention was modeled as a mediating construct linking motivational, contextual, and informational antecedents to actual usage behavior. Model fit was evaluated using the same fit indices applied in the measurement model.

All statistical tests were conducted using a significance level of *p* < 0.05.

**Figure 2:**
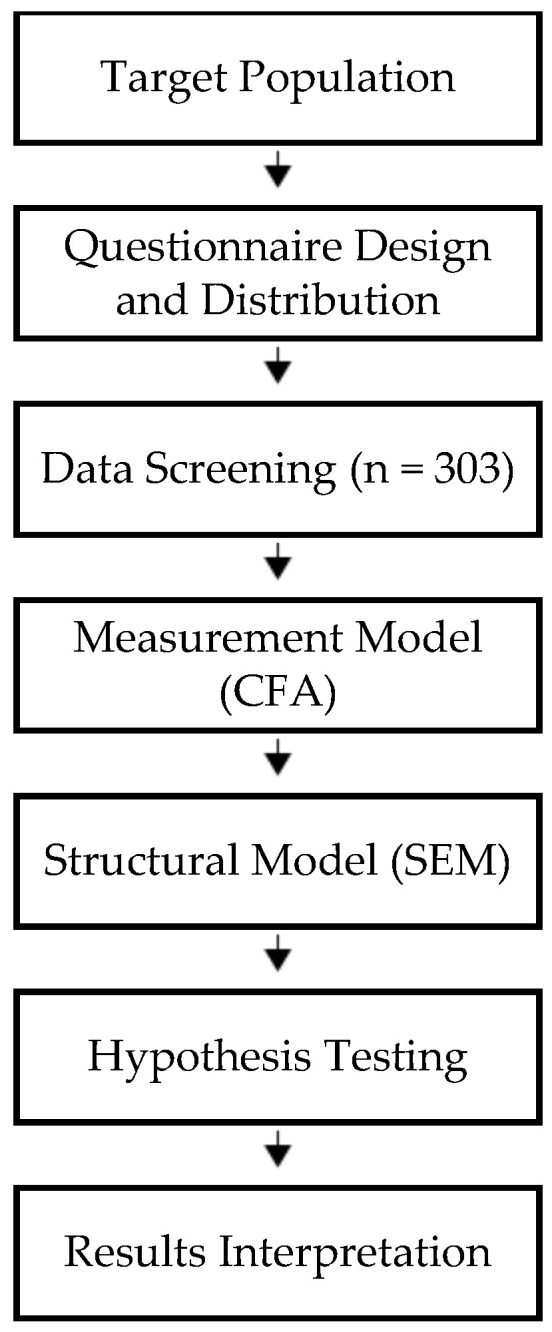
Research methodology flow diagram.

Figure 2 provides the research methodology flow diagram illustrating the sequential stages of the study, including target population identification, questionnaire distribution, data screening, measurement model assessment, structural model testing, and hypothesis evaluation.

## 4. Results

Before presenting the measurement and structural model results, the demographic characteristics of the participants are summarized. Table 3 presents the sample profile and nutritional supplement usage patterns among the respondents.

The sample consisted predominantly of participants from public universities (85%), while 15% were affiliated with foundation universities. Assistant professors represented the largest academic rank in the sample (29%), followed by lecturers (28%). Female participants accounted for 62% of the respondents. The largest age group was 35–44 years (42%). Among participants reporting current supplement use, vitamins and minerals were the most frequently consumed supplement categories.

Descriptive statistics and correlation coefficients among the study variables are presented in Table 4.

As shown in Table 4, the correlation coefficients among the study variables are generally positive and statistically significant, indicating meaningful associations and providing preliminary support for the proposed relationships.

To further examine potential differences across demographic groups, independent samples *t*-tests were conducted to compare the study variables by gender. The results are presented in Table 5.

As shown in Table 5, significant gender differences were observed for most variables, except for digital marketing influence (DMI), which did not differ significantly between groups.

In addition to gender differences, one-way ANOVA analyses were conducted to examine potential differences across age groups. The results are presented in Table 6.

As shown in Table 6, significant differences across age groups were observed for most variables, except for digital marketing influence (DMI), which did not vary significantly across age groups.

Post hoc comparisons (Tukey HSD) indicated that the significant differences were primarily observed between specific age groups.

### 4.1. Measurement Model

A confirmatory factor analysis (CFA) was performed to evaluate the psychometric properties of the measurement model prior to testing the structural paths. All latent variables were operationalized using multiple observed indicators. Model adequacy was assessed through several fit indices, including the chi-square to degrees of freedom ratio (χ^2^/df), Comparative Fit Index (CFI), Tucker–Lewis Index (TLI), and Root Mean Square Error of Approximation (RMSEA). The measurement model was initially specified by including all observed indicators associated with their respective latent constructs based on the theoretical framework. The initial CFA results indicated that model refinement was necessary. Following standard SEM procedures, the model was further evaluated to confirm indicator reliability and construct validity. The final measurement model retained all indicators that demonstrated satisfactory factor loadings and theoretical consistency, resulting in the model reported in this study.

To improve transparency, a comparison of the initial and final CFA model fit indices is presented in Table 7.

The results indicated that the measurement model demonstrated a reasonable level of fit to the data (χ^2^/df = 3.28; CFI = 0.878; TLI = 0.859; RMSEA = 0.087). Although some fit indices were slightly below the more conservative cut-off values suggested in the literature, the overall pattern of fit statistics indicates that the measurement model provides an adequate representation of the observed data [30,31,33].

All standardized item loadings were statistically significant and surpassed the recommended minimum value of 0.50, indicating adequate convergent validity. Composite reliability (CR) values exceeded the 0.70 threshold across all constructs, demonstrating strong internal consistency. Furthermore, most Average Variance Extracted (AVE) values met or surpassed the 0.50 criterion. Discriminant validity was assessed using the Fornell–Larcker criterion, whereby the square root of each construct’s AVE exceeded the correlations with other constructs, indicating satisfactory discriminant validity.

### 4.2. Structural Model and Hypothesis Testing

Following the validation of the measurement model, the structural model was estimated using structural equation modeling (SEM) to test the proposed hypotheses. The structural model also demonstrated a reasonable level of fit (χ^2^/df = 3.36; CFI = 0.872; TLI = 0.853; RMSEA = 0.089), indicating that the hypothesized relationships provide an adequate representation of the data. The structural relationships tested in the SEM analysis correspond to the conceptual framework presented in Figure 1.

The results indicate that health motivation had a strong and positive effect on usage intention (β = 0.736, *p* < 0.001), supporting H1. Academic work stress also demonstrated a positive effect on usage intention (β = 0.314, *p* < 0.001), supporting H2. In contrast, digital health literacy (β = 0.011, *p* > 0.05) and digital marketing influence (β = 0.022, *p* > 0.05) did not exhibit significant direct effects on usage intention; therefore, H3 and H4 were not supported.

Usage intention was found to be a strong and significant predictor of actual usage behavior (β = 0.85, *p* < 0.001), providing support for H5. Overall, the structural model results highlight the central role of motivational and contextual factors in shaping nutritional supplement usage intention, as well as the pivotal role of intention in translating these antecedents into actual usage behavior.

### 4.3. Summary of Hypothesis Testing

Table 8 presents the outcomes of the hypothesis tests, reporting standardized path estimates and their corresponding significance levels.

Overall, the results indicate that motivational and contextual factors significantly predict usage intention, whereas informational factors do not exhibit direct effects.

## 5. Discussion

This study set out to examine the behavioral determinants of nutritional supplement use among academics by integrating motivational, contextual, and informational factors within an intention–behavior framework. The findings provide clear evidence that intrinsic motivation and academic work stress play a central role in shaping usage intention, whereas informational factors such as digital health literacy and digital marketing influence do not exert direct effects in this highly educated consumer segment. In line with intention-based consumer behavior theories, usage intention emerged as a powerful predictor of actual usage behavior, underscoring the robustness of the intention–behavior link in health-related consumption contexts. Taken together, these results suggest that nutritional supplement use among academics is driven less by exposure to information or promotional stimuli and more by internally grounded motivational processes and situational pressures embedded in professional life. These findings directly address the research objective of the study, namely, to examine the behavioral determinants of nutritional supplement use among academics within an intention–behavior framework.

### 5.1. Health Motivation and Usage Intention

The strong positive effect of health motivation on usage intention highlights the central role of intrinsic drivers in health-related consumer behavior. Consistent with prior research emphasizing motivation as a key antecedent of preventive and self-care behaviors, the findings suggest that academics who are intrinsically motivated to maintain or enhance their well-being are more likely to develop intentions to use nutritional supplements. Unlike externally triggered consumption decisions, supplement use in this context appears to be guided by internally driven goals related to personal health management and self-regulation. This result reinforces motivation-based perspectives in consumer behavior literature, indicating that when consumption decisions are closely tied to individual well-being, intrinsic motivation outweighs informational or promotional influences in shaping intention. This centrality of intrinsic motivation aligns with evidence indicating that supplement consumption is largely shaped by preventive health orientation and habitual self-care practices [34], with motivated consumers adopting supplements as part of long-term well-being strategies [7].

Empirical evidence from different consumer contexts further supports the central role of health-oriented motivation in supplement adoption [35]. This motivational mechanism is consistent with prior population-level evidence indicating that individuals with greater health awareness and chronic health concerns are significantly more likely to adopt nutritional supplements as a form of preventive and compensatory health behavior [4]. For instance, Bayır et al. [36] reported widespread nutritional supplement use among Turkish consumers, with vitamins, minerals, and omega-3 products emerging as the most commonly consumed supplements. Importantly, their findings emphasized health consciousness and food safety knowledge as critical drivers of usage intention, underscoring the motivational foundations of supplement-related decision-making.

Similarly, Dong et al. [3] found that individuals with higher educational attainment and stronger health-oriented lifestyle patterns were significantly more likely to engage in nutritional supplement use. Their results further suggest that individuals who perceive their health status as suboptimal often adopt supplement consumption as compensatory health behavior. Together, these findings align closely with the present study, reinforcing the notion that intrinsic health motivation constitutes a primary mechanism underlying supplement-related intention across diverse demographic and cultural contexts. This centrality of intrinsic motivation is further supported by behavioral evidence showing that dietary supplement users tend to adopt supplements as part of broader preventive health lifestyles rather than in response to short-term needs or promotional exposure [2,37,38].

O’Dea [6] demonstrated that adolescents primarily consumed nutritional supplements due to perceived health and performance benefits, highlighting the role of intrinsic well-being motives rather than informational or promotional drivers—further supporting the central role of health motivation observed in the present study. This dominance of intrinsic drivers is consistent with prior behavioral evidence showing that personal attitudes toward supplements are the strongest predictors of consumption intention, outweighing external social and promotional influences [39]. Recent evidence further reinforces the central role of health-oriented psychological processes in shaping health-related consumption intention. Pan et al. [8] demonstrated that health consciousness exerts a significant indirect effect on purchase intention through sequential attitudinal and motivational mechanisms, indicating that internally driven health concerns translate into behavioral intention primarily via psychological engagement rather than external informational exposure. This supports the present study’s emphasis on intrinsic health motivation as the primary driver of nutritional supplement usage intention among academics.

### 5.2. Academic Work Stress and Usage Intention

The positive relationship between academic work stress and usage intention underscores the importance of contextual stressors in consumer decision-making. Academic work stress reflects sustained cognitive and emotional demands embedded in professional life, which may increase consumers’ perceived need for supportive or coping-related solutions. The findings suggest that nutritional supplements may be perceived as instrumental resources for maintaining performance, resilience, or balance under conditions of prolonged work-related stress. This extends prior consumer behavior research by positioning stress not merely as an outcome variable but as a meaningful antecedent of health-related consumption intention. In demanding occupational contexts, situational pressures may activate compensatory consumption motives, thereby increasing the likelihood of supplement adoption.

The tendency to adopt supplements as a response to fatigue and perceived physical strain echoes earlier findings showing that consumers frequently use supplements as compensatory tools to restore energy and improve functional performance [6]. Supporting this interpretation, Dong et al. [3] reported that individuals experiencing poorer sleep quality were more likely to use omega-3 and related supplements as a health-support strategy. Although their study did not directly examine occupational stress, this pattern suggests that supplement use may function as a compensatory response to physiological and psychological strain. This mechanism aligns closely with the present study’s findings, indicating that academic work stress serves as a contextual trigger that strengthens supplement-related usage intention [40].

This compensatory consumption pattern is consistent with broader lifestyle-based evidence suggesting that intensive work routines, sedentary behavior, and time scarcity motivate individuals to adopt nutritional supplements as practical health-support strategies [5].

### 5.3. Digital Health Literacy and Usage Intention

Contrary to expectations, digital health literacy did not exhibit a direct effect on usage intentions. While digital health literacy has been widely acknowledged as an important determinant of informed health decision-making [11,12], the present findings suggest that its role may be contingent on consumer characteristics. Among highly educated consumers such as academics, digital health literacy may function as a baseline capability rather than a differentiating factor in intention formation.

Consistent with dual-process models of information processing, individuals with higher cognitive resources tend to engage in more analytical evaluation, reducing the marginal influence of informational inputs on behavioral intention [19]. In this context, informational competence alone may be insufficient to motivate consumption unless accompanied by strong motivational or situational drivers. This finding contributes to consumer behavior literature by suggesting that the explanatory power of informational factors may diminish within segments characterized by high cognitive and educational resources.

### 5.4. Digital Marketing Influence and Usage Intention

Similarly, digital marketing influence did not directly predict usage intention. This finding challenges the assumption that promotional exposure universally drives consumer intention within digital health markets. Supporting this interpretation, Dong et al. [10] reported that despite increasing health awareness, many consumers remain skeptical about the effectiveness of nutritional supplements and the credibility of marketing claims. Such skepticism may function as a behavioral barrier that constrains the persuasive power of promotional communication, particularly among informed consumer groups.

Concerns regarding exaggerated health claims, regulatory ambiguity, and limited clinical evidence have also been identified as barriers to supplement adoption, reinforcing consumer skepticism toward promotional communication [5]. Consistent with this perspective, Gong et al. [4] noted that misleading advertising practices and limited professional guidance contribute to consumer distrust toward nutritional supplements, which, in turn, suppresses usage intention. Together, these findings suggest that promotional exposure alone is insufficient to directly drive intention when consumers actively evaluate credibility and perceived trustworthiness.

Complementing this evidence, Gabriels and Lambert [41] demonstrated that purchasing decisions were primarily shaped by peer recommendations and label-based safety information, while professional advice and usage instructions exerted comparatively limited influence. This pattern indicates that even in information-rich environments, consumers may privilege socially embedded and trust-related cues over direct promotional messages. Consistent with this interpretation, Nábrádi et al. [7] reported widespread consumer skepticism toward promotional claims in the dietary supplement market, emphasizing that trust and personal health beliefs play a far greater role than advertising in shaping consumption decisions.

Further insight into the nature of supplement marketing is provided by Hua et al. [42], who found that packaging-based promotional content frequently emphasizes aspirational outcomes, lifestyle associations, and persuasive framing rather than substantive product information. Such marketing strategies may generate symbolic appeal without enhancing informational value, particularly among cognitively sophisticated audiences. This further explains why promotional exposure may fail to directly translate into stronger usage intention. The lifestyle-oriented nature of supplement use further suggests that long-term health orientation may outweigh momentary promotional stimuli, as consumers who adopt supplements as part of routine wellness behaviors are less responsive to short-term marketing exposure [2].

For academically trained consumers, heightened persuasion knowledge and analytical processing may reduce susceptibility to superficial marketing cues. Rather than being passively influenced by promotional content, academics appear to rely more strongly on internally grounded judgment and motivational drivers when forming supplement-related intentions. This interpretation aligns with persuasion knowledge theory and dual-process models of information processing, suggesting that the effectiveness of marketing communications is contingent upon consumers’ cognitive sophistication and contextual characteristics.

### 5.5. Usage Intention and Actual Usage Behavior

Consistent with established intention–behavior frameworks, usage intention emerged as a strong predictor of actual usage behavior. This finding aligns closely with the Theory of Planned Behavior, which posits behavioral intention as the most proximal determinant of action [9]. Extensive empirical evidence has further demonstrated that stronger intentions substantially increase the likelihood of behavioral enactment across health-related contexts [10,22].

Despite concerns regarding intention–behavior gaps in certain health behaviors, the present results indicate that among academics, intention serves as a reliable mechanism linking motivational and contextual antecedents to actual usage. This underscores the robustness of intention-based models in explaining nutritional supplement consumption and highlights the importance of strengthening intention formation to influence real-world behavioral outcomes.

### 5.6. Theoretical Contributions

This study contributes to the literature by reframing nutritional supplement use among academics as a health-related consumption behavior shaped by the interaction of motivational, occupational, and informational determinants. Rather than treating supplement use merely as an individual health practice or a descriptive consumption pattern, the study positions it within an intention–behavior framework and demonstrates that intrinsic health motivation and academic work stress are more influential predictors of usage intention than digital health literacy and digital marketing influence. This finding highlights the boundary conditions under which informational and promotional factors may lose explanatory power among highly educated professional consumers.

Furthermore, the findings extend intention-based consumer behavior models by showing that intrinsic motivation and contextual stressors may play a more prominent role than informational or promotional factors within cognitively sophisticated consumer segments. This challenges the dominant emphasis on information availability and marketing exposure in health-related consumption research and provides a more nuanced understanding of the drivers of preventive health-related consumption behavior.

Second, by empirically establishing academic work stress as a significant antecedent of usage intention, this study contributes to consumer behavior research by positioning occupational stress as an active driver of health-related consumption decisions. While prior studies have largely examined stress as an outcome or moderator, the present findings highlight its role as a direct motivational force that shapes intention formation. This perspective broadens existing models of health consumption by integrating situational pressures embedded in professional life into the decision-making process.

Third, the non-significant effects of digital health literacy and digital marketing influence provide theoretical insight into the boundary conditions of informational determinants in consumer behavior models. Among highly educated consumers, informational competence may function as a baseline characteristic rather than a differentiating driver of intention. This finding refines existing theories by suggesting that the impact of informational and persuasive cues is contingent upon consumer characteristics, particularly education level and professional expertise.

Finally, the strong intention–behavior relationship observed in this study reinforces the robustness of intention-based frameworks in explaining nutritional supplement consumption. The findings indicate that once intention is established, it reliably translates into actual usage behavior, even in complex and credence-based consumption contexts. Collectively, these contributions advance consumer behavior theory by highlighting the primacy of motivational and contextual mechanisms over informational influences in shaping health-related consumption decisions among educated consumer segments.

### 5.7. Managerial and Practical Implications

The findings of this study offer several important practical implications for practitioners, policymakers, and digital health stakeholders involved in the nutritional supplement market. First, the strong role of health motivation suggests that interventions aimed at promoting supplement use should prioritize intrinsically framed messages that emphasize personal well-being, self-care, and long-term health management rather than relying solely on informational content. For highly educated consumer segments, communication strategies that align supplement use with individual health goals and lifestyle coherence are likely to be more effective than fact-heavy or purely promotional approaches.

Second, the significant effect of academic work stress on usage intention highlights the importance of contextualizing supplement consumption within occupational stress environments. Organizations, healthcare providers, and wellness platforms targeting professional populations may benefit from positioning nutritional supplements as complementary resources that support resilience, cognitive performance, and daily functioning under sustained work demands. Importantly, such positioning should be carefully framed to avoid medicalized or exaggerated claims and instead focus on responsible and supportive usage narratives.

Third, the non-significant effects of digital health literacy and digital marketing influence carry important implications for digital communication strategies. While investments in digital content and marketing campaigns remain widespread, the findings suggest that promotional intensity alone may not translate into intention among highly educated consumers. Practitioners should therefore move beyond exposure-driven metrics and focus on credibility, transparency, and alignment with consumers’ internal motivations. Influencer-based or promotional strategies may require greater emphasis on expertise, scientific grounding, and authenticity to maintain relevance within cognitively sophisticated audiences.

Finally, the strong intention–behavior linkage observed in this study underscores the importance of strategies that facilitate intention formation as a pathway to actual usage. Interventions that strengthen commitment—such as goal-setting tools, self-monitoring mechanisms, or personalized recommendations—may enhance the likelihood that favorable intentions are translated into consistent usage behavior. Overall, these implications suggest that effective engagement in the nutritional supplement market requires a nuanced understanding of consumer motivation and context, particularly when addressing educated professional segments.

## 6. Conclusions, Limitations, and Future Research

This study examined the behavioral determinants of nutritional supplement use among academics by integrating motivational, contextual, and informational factors within an intention–behavior framework. The findings demonstrate that intrinsic health motivation and academic work stress play a central role in shaping usage intention, whereas digital health literacy and digital marketing influence do not exert direct effects within this highly educated consumer segment. Moreover, usage intention was found to be a strong predictor of actual usage behavior, underscoring the robustness of intention-based models in explaining health-related consumption decisions. In addition, the findings reveal that demographic characteristics such as gender and age contribute to variations in the study variables. The additional analyses indicate that several constructs, including health motivation, academic work stress, and usage intention, differ significantly across demographic groups. These results highlight the importance of considering demographic heterogeneity when interpreting health-related consumption behaviors and further strengthen the robustness of the proposed model.

By focusing on academics as a distinct consumer group, this study contributes to consumer behavior literature by highlighting the importance of internal motivations and occupational context in health-related decision-making. The results suggest that nutritional supplement consumption among educated professionals is less responsive to external informational and promotional stimuli and more strongly guided by personal health goals and situational pressures embedded in professional life. In this respect, the study advances understanding of how consumer characteristics and context shape the relative influence of motivational and informational determinants.

Despite its contributions, this study is subject to several limitations that should be acknowledged. First, the cross-sectional research design limits the ability to draw causal inferences among the study variables. Future research may employ longitudinal or experimental designs to examine changes in supplement usage behavior over time. Second, the data were collected through self-reported measures, which may be subject to common method bias or social desirability effects. Incorporating objective usage data or mixed-method approaches could strengthen future investigations. Third, while the focus on academics offers valuable insights into a highly educated consumer segment, the generalizability of the findings to other professional or cultural contexts may be limited.

The use of a convenience sampling approach may further limit the generalizability of the findings. However, this approach was deemed appropriate given the study’s focus on academic professionals, a specific and relatively difficult-to-access population. Future research may benefit from employing probabilistic sampling techniques or examining more diverse populations to enhance the generalizability of the results.

Future research could extend the proposed model by examining additional psychological or contextual factors, such as trust in regulatory institutions, perceived scientific legitimacy, or social norms surrounding supplement use. Comparative studies across different occupational groups or cultural settings may further clarify the boundary conditions of the observed relationships. Overall, this study provides a solid foundation for future research on nutritional supplement consumption and underscores the importance of integrating motivation, context, and intention in consumer behavior models.

## Figures and Tables

**Table 1:** Measurement scale sources and adaptation process.

Construct	Items	Adapted from	Adaptation Rationale
Health Motivation (HM)	4	Moorman & Matulich, 1993 [14]	Items were adapted to capture academics’ intrinsic motivation to maintain and improve health through nutritional supplement use.
Academic Work Stress (AWS)	4	Lazarus & Folkman, 1984 [15]; Duhachek, 2005 [26]	Stress-related items were contextualized to reflect academic workload, fatigue, and work-related strain.
Digital Health Literacy (DHL)	4	Norman & Skinner, 2006 [11]; van der Vaart & Drossaert, 2017 [27]	Items were adapted to assess the ability to evaluate digital health information about nutritional supplements.
Digital Marketing Influence (DMI)	4	Dehghani & Tumer, 2015 [13]; Lou & Yuan, 2019 [29]	Items were adapted to reflect the perceived influence of online reviews, recommendations, influencers, and digital promotional content on supplement-related decisions.
Usage Intention (UI)	4	Ajzen, 1991 [9]	Items were adapted to measure intention to use nutritional supplements in the near future and under relevant health/work conditions.
Actual Usage Behavior (AUB)	4	Ajzen, 1991 [9]	Items were adapted to assess self-reported actual supplement use frequency and consistency.

Note: Measurement items were adapted from previously validated scales.

**Table 2:** Measurement model results.

Construct	CR	AVE
HM	0.93	0.76
AWS	0.79	0.49
DHL	0.70	0.44
DMI	0.83	0.56
UI	0.89	0.68
AUB	0.84	0.64

**Table 3:** Sample characteristics and supplement usage profile.

Variable	Category	*n*	%
Institution Type	Public University	257	85
	Foundation University	46	15
Academic Title	Professor	37	12
	Associate Professor	48	16
	Assistant Professor	87	29
	Lecturer	84	28
	Research Assistant	47	15
Gender	Female	189	62
	Male	114	38
Age Group	25–34	71	23
	35–44	126	42
	45–54	84	28
	55–64	19	6
	65 and over	3	1
Nutritional Supplement Type Used *	Vitamins	201	87
	Minerals	168	73
	Protein supplements	75	33
	Cognitive/mental performance supplements	72	31
	Herbal supplements	61	26
	Energy/stress/sleep supplements	21	9
	Sports performance supplements	20	8

Notes: * Percentages for supplement types may exceed 100% as participants were allowed to report multiple supplement categories. Supplement usage frequencies are reported for participants who indicated current supplement use (regular or occasional; *n* = 231).

**Table 4:** Descriptive statistics and correlations.

Variable	Mean	SD	1	2	3	4	5	6
HM	3.3086	1.1063	1					
AWS	3.9439	0.7249	0.568 **	1				
DHL	3.6287	0.4999	0.280 **	0.306 **	1			
DMI	2.4777	0.9976	0.352 **	0.316 **	0.072	1		
UI	3.5223	1.1337	0.852 **	0.597 **	0.249 **	0.355 **	1	
AUB	3.3003	1.1315	0.816 **	0.584 **	0.272 **	0.303 **	0.849 **	1

Notes: N = 303. ** *p* < 0.01.

**Table 5:** Differences in study variables by gender.

Variable	Female Mean	Male Mean	t	*p*
DHL	3.6865	3.5329	2.616	0.009
DMI	2.4762	2.4803	−0.034	0.973
HM	3.4339	3.1009	2.453	0.015
AWS	3.8849	3.2829	5.322	0.000
UI	3.6720	3.2741	2.901	0.004
AUB	3.4352	3.0768	2.621	0.009

**Table 6:** Differences in study variables by age groups (ANOVA).

Variable	F	*p*
DHL	2.433	0.048
DMI	0.197	0.940
HM	4.353	0.002
AWS	5.087	0.001
UI	3.271	0.012
AUB	2.793	0.027

**Table 7:** Comparison of initial and final CFA models.

Fit Index	Initial CFA Model	Final CFA Model
χ^2^/df	5.089	3.280
CFI	0.662	0.878
TLI	0.631	0.859
RMSEA	0.116	0.087

**Table 8:** Summary of hypothesis testing results.

Hypothesis	Path	Std. β	Result
H1	HM → UI	0.736	Supported
H2	AWS → UI	0.314	Supported
H3	DHL → UI	0.011	Not supported
H4	DMI → UI	0.022	Not supported
H5	UI → AUB	0.85	Supported

Notes: HM = Health Motivation; AWS = Academic Work Stress; DHL = Digital Health Literacy; DMI = Digital Marketing Influence; UI = Usage Intention; AUB = Actual Usage Behavior.

## Data Availability

The data presented in this study are available on request from the corresponding author due to privacy and ethical restrictions related to participant confidentiality.

## Appendix A. Measurement Items

**Health Motivation (HM)**

HM1: I consider using nutritional supplements to maintain my health.

HM2: I am motivated to take preventive actions to improve my health.

HM3: I believe that using supplements contributes to my overall well-being.

HM4: Maintaining my health is an important priority in my daily life.

**Academic Work Stress (AWS)**

AWS1: My academic workload makes me feel physically exhausted.

AWS2: I often feel stressed due to academic responsibilities.

AWS3: Long working hours negatively affect my health.

AWS4: I feel that stress reduces my overall well-being.

**Digital Health Literacy (DHL)**

DHL1: I can evaluate the reliability of health-related information found online.

DHL2: I feel confident in distinguishing accurate from inaccurate health information on the internet.

DHL3: I can effectively use online resources to make health-related decisions.

DHL4: I understand health-related information available on digital platforms.

**Digital Marketing Influence (DMI)**

DMI1: Online reviews influence my decisions about using nutritional supplements.

DMI2: Social media content affects my perception of supplements.

DMI3: Recommendations from influencers impact my supplement choices.

DMI4: Digital advertisements affect my intention to use supplements.

**Usage Intention (UI)**

UI1: I intend to use nutritional supplements in the near future.

UI2: I am likely to use supplements when needed.

UI3: I plan to use supplements regularly.

UI4: I will consider using supplements to improve my health.

**Actual Usage Behavior (AUB)**

AUB1: I currently use nutritional supplements.

AUB2: I use supplements regularly.

AUB3: I frequently consume nutritional supplements.

AUB4: I have used supplements in recent periods.
